# Contribution of Murine Models to the Study of Malaria During Pregnancy

**DOI:** 10.3389/fmicb.2019.01369

**Published:** 2019-06-19

**Authors:** André Barateiro, Marcelo L. M. Pereira, Sabrina Epiphanio, Claudio R. F. Marinho

**Affiliations:** ^1^Department of Parasitology, Institute of Biomedical Sciences, University of São Paulo, São Paulo, Brazil; ^2^Institute of Biosystems and Integrative Sciences, Faculty of Sciences, University of Lisbon, Lisbon, Portugal; ^3^Department of Clinical Analysis and Toxicology, Faculty of Pharmaceutical Sciences, University of São Paulo, São Paulo, Brazil

**Keywords:** murine models, *Plasmodium* spp., malaria, pregnancy, placental malaria

## Abstract

Annually, many pregnancies occur in areas of *Plasmodium* spp. transmission, particularly in underdeveloped countries with widespread poverty. Estimations have suggested that several million women are at risk of developing malaria during pregnancy. In particular cases, systemic infection caused by *Plasmodium* spp. may extend to the placenta, dysregulating local homeostasis and promoting the onset of placental malaria; these processes are often associated with increased maternal and fetal mortality, intrauterine growth restriction, preterm delivery, and reduced birth weight. The endeavor to understand and characterize the mechanisms underlying disease onset and placental pathology face several ethical and logistical obstacles due to explicit difficulties in assessing human gestation and biological material. Consequently, the advent of murine experimental models for the study of malaria during pregnancy has substantially contributed to our understanding of this complex pathology. Herein, we summarize research conducted during recent decades using murine models of malaria during pregnancy and highlight the most relevant findings, as well as discuss similarities to humans and the translational capacity of achieved results.

## Malaria in Human Pregnancy

Malaria still represents a serious public health issue for several communities distributed worldwide. Estimations from 2017 have shown that at least 219 million cases occurred in areas of *Plasmodium* spp. transmission, in which approximately half a million victims died from the disease (World Health Organization, 2018). These estimations encompass pregnant women, who are particularly more susceptible to developing severe clinical manifestations resulting from malaria in pregnancy (MiP) induced by *Plasmodium* spp. Although outdated, estimations performed by Dellicour et al. (2010) noted 125.2 million pregnancies occurring in malaria-endemic areas. This study came as a follow-up of previous estimations suggesting that 25 million pregnancies occur in areas of *P. falciparum* transmission alone, distributed across sub-Saharan Africa (Desai et al., 2007). Nevertheless, current epidemiologic knowledge is inaccurate and imprecise and might support outdated and underestimated predictions, hiding a much more alarming reality.

This concerning epidemiological scenario highlights the importance of conducting preventive measures to control MiP, which might drastically evolve to placental malaria (PM), a pathology frequently associated with the occurrence of poor outcomes during pregnancy, such as maternal and fetal mortality, intrauterine growth restriction (IUGR), preterm birth and reduced birth weight (Desai et al., 2007; Umbers et al., 2011; Rogerson et al., 2018). These deleterious consequences affecting both the mother and the growing fetus are reflections of histological and physiological changes occurring within the placenta [recently summarized elsewhere (Sharma and Shukla, 2017)]. One of the key features of *P. falciparum* PM is the accumulation of *P. falciparum*-infected erythrocytes (IE) in the placenta (Beeson et al., 2002; Muthusamy et al., 2004). Upregulation of the VAR2CSA protein, a variant of the highly polymorphic adhesion peptide *P. falciparum* erythrocyte membrane protein 1 (PfEMP1) (Salanti et al., 2003), is responsible for parasite sequestration upon preferential binding to chondroitin sulfate A (CSA), which is abundantly expressed in the placenta (Fried et al., 2006; Muthusamy et al., 2007). Consequently, a severe local inflammatory process is triggered, characterized by the infiltration of monocytes and leukocytes in the placenta and eventual inflammation in response to parasite accumulation (Ismail et al., 2000; Parekh et al., 2010; Lucchi et al., 2011; Souza et al., 2013). This process has been frequently associated with placental histological alterations during MiP, such as dysregulation of placental architecture, formation of syncytial knots, fibrin deposition, necrosis, and placental barrier thickening (Walter et al., 1982; Ismail et al., 2000; Souza et al., 2013), and has been reported to occur during IUGR and preterm delivery, as well as in cases of reduced birth weight (Moormann et al., 1999; Menendez et al., 2000; Rogerson et al., 2003; Umbers et al., 2011).

Although we have obtained substantial knowledge in the field, studies on the epidemiology and pathology of MiP are frequently challenging due to related ethical and logistic difficulties. Long gestational periods, uncontrollable experimental planning, difficult access to biological and placental samples, and critical constraints associated with human experiments represent significant barriers that slow research progress and the understanding of this severe and complex disease. Therefore, alternatives have emerged with the advent of murine models to study MiP that have brought fundamental knowledge to the field. Herein, we have compiled research conducted for the past four decades using experimental rodent models, highlighting the most relevant findings, similarities to humans and, consequently, the translational capacity of achieved results.

## Human and Murine Gestation: Comparison and Translational Challenges for the Study of MiP

### Comparative Gestation Development

In parallel with some other features, gestation length and development are somewhat different between human and murine mammals. Human gestation lasts for approximately 38 weeks (three trimesters) in contrast to rodents, in which gestation takes place over a 3-week period (Murray et al., 2010). In addition, there are also slight differences between species regarding the implantation period. Murine implantation takes place between the fourth and fifth days of gestation, somewhat sooner than in humans, in which this phenomenon occurs between the fifth and sixth days (Rossant and Tam, 2017). After implantation, gestation will progress toward a shift in fetal nutritional means. During early gestation in both humans and rodents, the growing fetus will survive through means of histiotrophic nutrition, in which nutrients are acquired by the uptake of substances secreted from the uterine glands present in the endometrium (Burton et al., 2002; Georgiades et al., 2002). However, the nutritional strategy changes around mid-gestation when the maternal blood supply to the placenta is completely established. Accordingly, the nutrient and gas exchanges between the mother and the fetus become functional, marking the onset of placental hemotrophic nutrition (Burton et al., 2001; Georgiades et al., 2002). In humans, this phenomenon occurs between the end of the first and the beginning of the second trimester, while in mice and rats, the same scenario occurs specifically at mid-gestation (between the twelfth and thirteenth days) (Georgiades et al., 2002). This shift in nutritional strategy occurs with the onset of organogenesis and fetal development, after which growth will continue until gestational term is reached.

### Comparative Placental Function, Structure, and Histology

Human and murine placentas have a considerable degree of similarity and are nearly identical from physiological and functional perspectives (Rossant and Cross, 2001; Georgiades et al., 2002). In both mammalian species, this transient organ ensures nutrient and gas exchange between the mother and the growing fetus (Lager and Powell, 2012), maintains tolerance to the maternal immune system (Kanellopoulos-Langevin et al., 2003), and works as a physical and immunological barrier against endogenous pathogens (Robbins and Bakardjiev, 2012).

**FIGURE 1:**
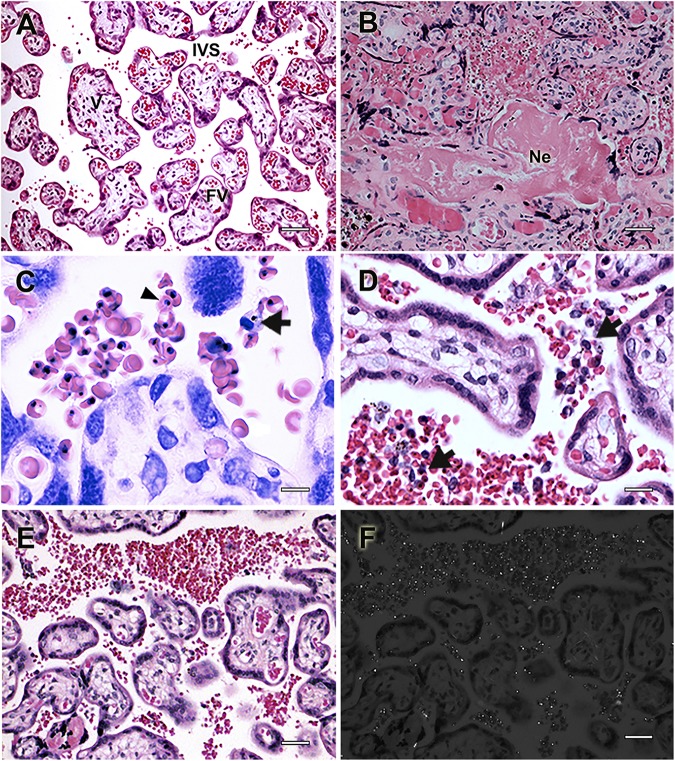
Histologic events associated with placental pathology during human malaria in pregnancy. Placental histologic sections of non-infected **(A)** and *P. falciparum*-infected **(B–F)** women stained with Hematoxylin-Eosin. **(A)** Healthy human placenta without tissue damage and normal architecture in which intervillous spaces (IVS), usually bathed in maternal blood are in contact with placental villi (V), feeding the growing fetus through the fetal vessels (FV). **(B)** *P. falciparum*-infected placentas often characterized by extensive necrotic areas (Ne) and tissue disorganization. **(C,D)** Mononuclear cells infiltrate (arrow) is frequently patent due to the accumulation of parasitized erythrocytes (arrowhead). **(E)** Massive sequestration of *P. falciparum*-infected erythrocytes in the placenta is accompanied by **(F)** the accumulation of hemozoin in the IVS, which can be detected by polarized light microscopy. Scale bars represent **(A,B,E,F)** 100 μm, **(C)** 5 μm, and **(D)** 15 μm. Microscopy pictures were taken by Rodrigo Medeiros de Souza.

**FIGURE 2:**
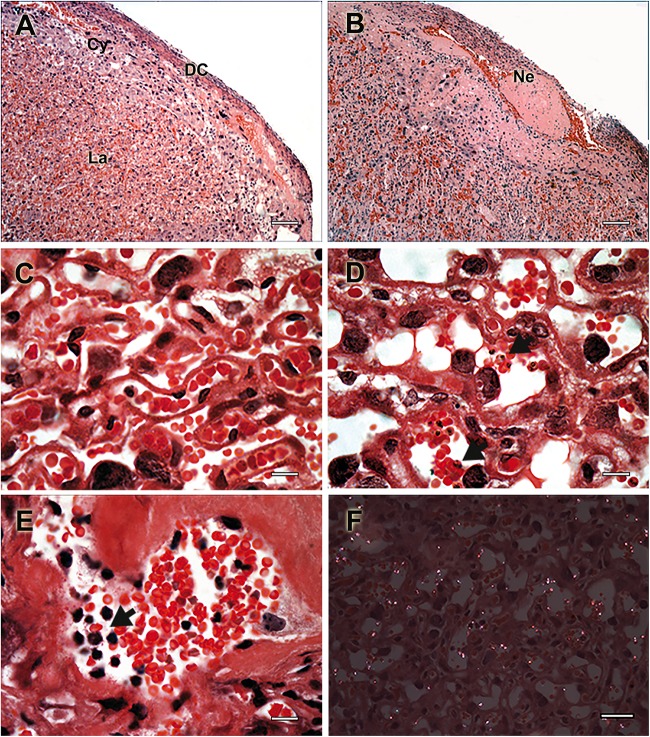
Histologic events associated with placental pathology during murine malaria in pregnancy. Placental histology of non-infected **(A,C)** and *P. berghei*-infected **(B,C,E,F)** mice stained with Hematoxylin-Eosin. **(A)** Healthy placenta with normal histologic structure characterized by normal distribution of layer-specific cell such decidual cells (DC), trophoblastic cells (Cy), and labyrinthic cells (La), having **(C)** a normal labyrinth organization. **(B)** Fibrinoid necrosis (Ne) is depicted in section of *P. berghei*-infected placentas with **(D)** massive tissue disorganization and trophoblast membrane thickening. **(E)** Mononuclear cell infiltrate is visible (arrow), which occurs in response to **(D)** parasite accumulation (arrow) and **(F)** hemozoin deposition observed using polarized light microscopy. Scale bars represent **(A,B)** 100 μm, **(C–E)** 15 μm, and **(F)** 30 μm. Figure was modified from Neres et al. (2008) and Marinho et al. (2009) upon permission granted on behalf of journal’s editorial board.

**FIGURE 3:**
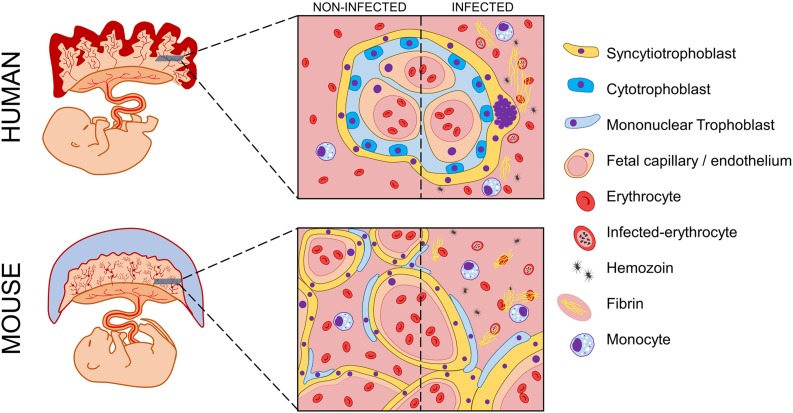
Schematic representation of possible histologic events in human and murine placentas during malaria in pregnancy. Human and murine fetuses are represented with corresponding placentas. Magnification of transversal slices of each placental core illustrate histologic architecture, which widely differs between human placental villi and murine labyrinthine structure. During malaria in pregnancy, both human and murine placentas experience histopathologic manifestations that impair local homeostasis and lead to poor pregnancy outcomes. A comparison between both infected placentas and their non-infected counterparts is depicted. The presence of monocyte infiltrate is visible, which occurs in response to parasite and hemozoin accumulation. Histologic modifications such as fibrin deposition and syncytial knots occur due to the onset of severe local inflammation. Legend of picture components is vertically displayed beside the scheme.

The placentas in both species are discoid organs in which maternal blood coming from the endometrium circulates and is in direct contact with trophoblasts, the fetal-derived cells responsible for regulating most placental physiological functions. In both species, the placenta can be structurally divided into three main areas: (1) an outer layer, consisting of uterine decidual cells and maternal blood vessels; (2) a middle layer, considered the implantation site where the placenta attaches to the uterus; and (3) an inner layer, in which the maternal blood interacts with trophoblasts to promote vital metabolic exchanges [reviewed elsewhere (Georgiades et al., 2002)]. Accordingly, the outer area can be mainly described as being composed by the myometrium and decidua basalis, which are extensively infiltrated by maternal arteries. This area is partially invaded by fetal-derived trophoblasts during the implantation process, which is considerably similar between both mammalian species. Moving toward the inner area of the human placenta, it is possible to distinguish a structural layer with no counterpart in the murine placenta known as the basal plate. This implantation site contains several distinct trophoblast subpopulations, such as extravillous (EVT) and cytotrophoblast (CT) cells. Nevertheless, an analogous area defined as the junctional zone occurs in murine animals, which is populated by specific types of cells such as trophoblast giant cells (TGC) and spongiotrophoblasts (SPG). Inside the placenta, more pronounced differences are observed regarding morphology and structure. The central area, which is commonly referred to as the human fetal placenta, is constituted by villi in an extremely ramified tree-like structure, which increases the contact surface with the maternal blood freely circulating within the intervillous space (IVS) (Figure 1A). On the other hand, a similar structure known as the labyrinth develops in mice and rats (Figures 2A,C). This impacts maternal blood circulation inside the placenta, where blood stays confined to the tortuous and sinusoidal channels (Rossant and Cross, 2001). Additionally, the surface area contacting the maternal blood is somewhat distinguished between humans and rodents from both cytological and structural perspectives. In the human placenta, maternal blood is separated from fetal capillaries by a single layer of syncytiotrophoblasts (ST), beyond which CT cells are present together with the basal lamina and fetal endothelium (schematically represented in Figure 3). According to these characteristics, the human placenta is classified as hemomonochorial (Takata et al., 1997; Georgiades et al., 2002). In opposition, three layers compose the murine labyrinthine wall: one composed of mononuclear trophoblasts dispersed throughout the surface contacting maternal blood and two ST layers definitively separating the maternal and fetal compartments (illustrated in Figure 3). Accordingly, murine placentas are classified as hemotrichorial (Takata et al., 1997).

Nevertheless, the structural divergences of human and murine placentas have occurred in parallel with an extensive variety of resident cell populations that are independent of their analogs but not similar in development and characteristics and are equally responsible for the same physiological and functional processes in human, mouse and rat placentas (Figure 3).

### Using Murine Models to Study Malaria in Human Pregnancy

It is required that a suitable experimental model includes a group of features that lead to valid and translatable findings. As such, it is pivotal that rodent physiology and genetics, as well as pathologic manifestations during disease, resemble those in humans to a certain extent, ultimately validating them as models for human research (Justice and Dhillon, 2016). Accordingly, after several models were reviewed, mice and rats were validated by Desowitz as suitable to study MiP (Duffy and Fried, 2001) under the assumption that physiological and pathological similarities observed between rodents and humans were sufficient to consider them appropriate to study this disease. Shared placental characteristics, such as hemochorial and discoid structure (Georgiades et al., 2002; Wildman et al., 2006), hemotrophic nutrition (Burton et al., 2001; Georgiades et al., 2002) and analogous placental cell populations (Rossant and Cross, 2001; Georgiades et al., 2002), encourage their usage as models of MiP. These similarities are supported by molecular phylogenetic analysis, which clusters both rodents and humans into the same evolutionary clade (Wildman et al., 2006). The molecular similarities can be extended to immunity, an important aspect to consider when studying a disease in which poor outcomes are predominantly dependent on the host’s immune response to malaria (Moormann et al., 1999; Rogerson et al., 2003). Hence, despite controversial discussion, it was shown that patterns of gene expression and biological pathways altered in mouse models of inflammatory diseases were significantly correlated with those from corresponding human conditions (e.g., sepsis) (Takao and Miyakawa, 2015), further supporting the usage of these animals for studying inflammatory diseases such as MiP. Together with the features mentioned above, the short gestational period, capacity for frequent and successive pregnancies, as well as large litter sizes, allow the rapid gathering of a considerable number of biological samples, which makes the rodents a powerful model for studying any pregnancy-related disease.

In addition to host characteristics, parasite intrinsic features are essential to validate experimental models of MiP. Above all, the most relevant features to consider should be the similarities between human and murine parasites cytoadhesion mechanisms. It is well known that the pathogenesis mechanisms involved in severe malaria induced by *P. falciparum* are associated with tissue- and organ-specific sequestration of IE (Schofield and Grau, 2005). More specifically, *P. falciparum* MiP may lead to severe PM as a result of IE sequestration in the placenta, which is mediated by the PfEMP1 variant VAR2CSA (which binds to placental CSA). The wide range of PfEMP1 variants known to be encoded by 50–60 *var* genes (Kraemer and Smith, 2006) have no known homologs in murine parasites (Hall et al., 2005). In fact, only some genetic signatures are similar between the murine and human *Plasmodium* species that encode alternate variable surface antigens (VSA), which were grouped in the multigenic *pir* superfamily (*Plasmodium* interspersed repeats) (Janssen et al., 2004; Hall et al., 2005). Nevertheless, murine parasite strains such as *P. berghei* have been shown to accumulate in specific tissues such as brain, fat, lung and spleen in a CD36-dependent and independent manner, which is also a well conserved pathogenesis mechanisms of severe malaria induced by *P. falciparum* (Franke-Fayard et al., 2010). In the same extent, the expression of alternate VSA and the binding capacity to placental CSA observed in murine parasites support the conclusions regarding the similarities between the pathogenesis mechanisms involved in murine and human disease (Hall et al., 2005; Neres et al., 2008; Marinho et al., 2009; Hviid et al., 2010). Distinct mechanisms of pathogenesis might also be directly linked with the biology of the parasites. As such, it is advisable to consider that the usage of murine parasites needs to be adjusted with caution since specific strains might better mimic the distinct diseases associated with the unrelated biology of human parasites (e. g. *P. falciparum* or *P. vivax*). Accordingly, disease severity in mice tends to be higher in *P. berghei* ANKA causing lethal anemia and cerebral malaria in C57BL/6 for instance (resembling *P. falciparum* in humans); however, it has a tropism for reticulocytes (similar to *P. vivax*). Differently, *P. chabaudi* invades mature erythrocytes and is responsible for a less severe pathology shown by the resistance to infection observed in BALB/c and C57BL/6 mice. In the same extent, different parasites might also have different tendencies to accumulation in specific tissues, being better used to study specific diseases and host-pathogen interactions (reviewed in Lamb et al., 2006).

Despite the aforementioned host and parasite characteristics, one must carefully translate findings obtained in murine models to humans, especially regarding preclinical observations, due to the observed differences between both mammals. Nevertheless, the following sections will depict some of the current knowledge acquired using murine models of MiP (research details for each model are presented in Table 1) during the past decades while describing the parallel characteristics existing between humans and rodents in regard to poor pregnancy outcomes and MiP placental pathology (summarized list in Table 2).

**TABLE 1:** Summary of the principal findings and observations done using murine models of malaria in pregnancy.

**Author(s)**	***Plasmodium* strain**	**Murine strain**	**Recrudescence**	**Anemia**	**Maternal mortality**	**Abortion Resorption Stillbirth Preterm delivery**	**Litter size Newborn/fetal weight/health**	**Placental abnormalities/alterations**
van Zon and Eling,1980	*P. berghei* K173	Swiss Albino	↑ Primigravidae	X	↑ In recrudescence	X	X	X
		C3H/StZ	↓multigravidae		↑ primigravidae			
		BALB/c	↑ due to					
		B10LP	↓immunity					

van Zon et al.,1982	*P. berghei* K173	Swiss Albino	→ To pregnancy	X	↑ In recrudescence	Preterm delivery ↑ in recrudescence	X	X

Oduola et al.,1982	*P. berghei*	A/J	→ To pregnancy	↑ In pregnancy	↑ In early infection	Stillbirth → to early infection	Normal litter size	Impaired labyrinth structure
	NK65	ICR			↓ in late infection		↓ Weight → to late infection	Trophoblast barrier thickening
					↑ parturition in late infection		↑ spleen weight	IEs/Hz
							No congenital malaria	MΦ

van Zon et al.,1985	*P. berghei*	Swiss Albino	→ To pregnancy	X	X	X	X	X
	K173	B10LP						

Vinayak et al.,1986	*P. berghei* NICD	Swiss Albino	X	X	↑ In early infection	Stillbirth → to mid gestation infection	↓ Litter size due to resorption	Hyperplasia
					Death before parturition	↑ resorption	↓ weight	↓ placental sinusoids
							↑ Spleen weight	IEs/Hz
							No congenital malaria	

Oduola et al.,1986	*P. berghei*	Sprague-Dawley	X	X	X	X	X	Hyperplasia
	NK65	ICR						Necrosis/fibrin
								Impaired labyrinth structure
								Trophoblast barrier thickening
								IEs/Hz
								MΦ/Leu

Desowitz et al.,1989	*P. berghei*	Wistar	No recrudescence	↑ In pregnancy	↑ In early infection	No preterm delivery	Normal litter size	↑ Placental parasitemia
	NYU-2				↓ in mid gestation infection with ↑ parasitemia at term			

Hioki et al.,1990	*P. berghei*	BALB/c	X	↑ In pregnancy	↑ In early infection (death before term)	X	X	X
	NK65			↑ in mid gestation infection	↓ in late infection (live until term)			

Pathak et al.,1990	*P. berghei*	Swiss Albino	No recrudescence	X	↑ Maternal mortality	Stillbirth → to mid gestation infection	↓ Weight	Hyperplasia
	NICD	VRC			↓ in treated pregnant mice		↓ Litter size	IEs

Pavia and Niederbuhl,1991	*P. yoelii* YM	ICR	No recrudescence	X	↑ Maternal mortality in non-immunized pregnancies	X	Delivery of dead pups in non-immunized pregnancies	X
	*P. yoelii* 17X				↓ with ↓ parasite virulence		No congenital malaria	

Tegoshi et al.,1992	*P. berghei*	Wistar	X	X	X	X	X	Hyperplasia
	NYU-2							Necrosis/fibrin
								Impaired labyrinth structure
								Trophoblast barrier thickening
								↑ IEs after late infection
								MΦ/Leu

Adachi et al.,2000	*P. berghei*	BALB/c	X	X	X	X	Congenital malaria	X
	ANKA							

Poovassery and Moore,2006	*P. chabaudi*	C57BL/6	X	↑ In pregnancy → to	↑ Mortality → pregnancy	↑ Abortion/resorption at mid gestation → to early infection	↓ Fetal viability	↑ IEs at mid gestation
	AS			↑ parasitemia				↓ monocyte accumulation

Neres et al.,2008	*P. berghei*	BALB/c	X	X	↑ Mortality → pregnancy	↑ Abortion/resorption during infection	↓ Fetal/birth weight	Hyperplasia
	ANKA					↑ preterm delivery during infection	↓ fetal blood flow/content	Necrosis/fibrin
							↓ viability	Impaired labyrinth structure
								Trophoblast barrier thickening
								IEs/Hz
								MΦ
								↓ Placental vasculature

Poovassery and Moore,2009	*P. chabaudi*	C57BL/6	X	X	X	↑ Abortion at mid gestation → to early infection	↓ Fetal viability	IEs/Hz
	AS							

Poovassery et al.,2009	*P. chabaudi*	C57BL/6	X	↑ In pregnancy → to	↑ Mortality → pregnancy	↑ Abortion/resorption at mid gestation → to early infection	↓ Fetal viability → to IFN-γ/TNF-α	Fibrin thrombi
	AS			↑ parasitemia				Placental hemorrhage
								Impaired labyrinth structure
								Monocytes/macrophages

Megnekou et al.,2009	*P. berghei*	BALB/c	→ To pregnancy	→ To recrudescence	→ To recrudescence	X	→ To recrudescence	iRBCs/Hz
	K173		↑ primigravidae		↑ due to ↓ immunity		↓ Litter size	
							↓ Weight	

Marinho et al.,2009	*P. berghei*	BALB/c	→ To pregnancy	X	→ To recrudescence	X	→ To recrudescence	Trophoblast barrier thickening
	ANKA				↑ primigravidae		↓ Litter size	IEs
					↓ multigravidae		↓ Weight	MΦ/Tc/NKc
							↓ Primigravidae	↓ Placental vascular spaces
							↑ Multigravidae	

Silver et al.,2010	*P. berghei*	BALB/c	X	X	X	↑ Abortion/resorption at late-gestation → to mid gestation infection	↓ Fetal weight at late gestation	X
	ANKA						↓ fetal viability	

Sarr et al.,2012	*P. chabaudi*	A/J	X	↑ In pregnant A/J mice when compared to C57BL/6	↑ In pregnant A/J mice when compared to C57BL/6	↑ Abortion/resorption in A/J and C57BL/6	X	↑ Placental IEs in A/J when comparing to C57BL/6
	AS	C57BL/6						

Rodrigues-Duarte et al.,2012	*P. berghei*	C57BL/6	X	X	↑ In pregnant mice infected with *P. berghei* NK65	↑ Stillbirth → mid gestation infection	↓ Fetal weight at late gestation	Necrosis/fibrin
	K173						↓ newborn viability	Impaired labyrinth structure
	NK65							Trophoblast barrier thickening
	ANKAΔpm4							IEs
								↓ Placental vascular spaces

Avery et al.,2012	*P. chabaudi*	C57BL/6	X	↑ In pregnancy → to	X	↑ Abortion at mid gestation → to early infection → to coagulation and impaired fibrinolysis	↓ Fetal viability	Necrosis/fibrin
	AS			↑ parasitemia				Impaired labyrinth structure

Conroy et al.,2013	*P. berghei*	BALB/c	X	X	X	X	↑ Fetal weight and ↑ viability in infected C5aR^–/–^ mice	↑ Placental vascular remodeling in infected C5aR^–/–^ mice
	ANKA	Wildtype						
		C5aR^–/–^						

de Moraes et al.,2013	*P. berghei*	BALB/c ♀	X	X	X	X	X	Impaired labyrinth structure
	ANKA	C57BL/6 ♂						Trophoblast barrier thickening
								Placental vasculature remodeling
								IEs in ↓ blood flow areas

Barboza et al.,2014	*P. berghei*	C57BL/6	X	X	X	X	↑ Fetal weight in infected MyD88^–/–^ mice	↑ Placental vascular spaces in infected MyD88^–/–^ mice
	NK65	Wildtype					↑ survival in newborn from infected MyD88^–/–^ mice	
		MyD88^–/–^						

Sharma and Shukla,2014	*P. berghei*	BALB/c	X	X	↓ In pregnant-infected mice treated with CQ/SP	X	↑ Weight and ↑ viability in newborn from mice treated with CQ/SP	↓ IEs, ↓ Hz, and normal placental histology in mice treated with CQ/SP
	NK65							

Lima et al.,2014	*P. berghei*	C57BL/6	X	X	X	X	X	Accumulation of iRBCs mature forms in the placenta
	*P. chabaudi*							IEs uptake by trophoblast

Sarr et al.,2015	*P. chabaudi*	A/J	X	X	X	↑ Abortion/resorption in A/J and C57BL/6	X	↑ Tc, Bc, NKc in conceptus from infected A/J and C57BL/6 mice
	AS	C57BL/6						↑ MΦ in the placental junctional zone of A/J mice
								Apoptosis present in immune cells and spongiotrophoblast

Sharma et al.,2016	*P. chabaudi*	C57BL/6	→ To pregnancy	→ To recrudescence	↑ In reinfections but not in recrudescence	↑ Stillbirth in reinfections and → to high parasitemia	Normal litter size	IEs in recrudescence
	CB		→ to CB sporozoites	→ to reinfection			↓ weight and ↓ malaria susceptibility in reinfection	No histologic alterations
	*P. chabaudi*							
	AS							

Barboza et al.,2017	*P. berghei*	C57BL/6	X	X	X	X	↑ Fetal weight in infected TLR4^–/–^ mice	IEs
	NK65	Wildtype						MΦ/NΦ/Dc
		TLR2^–/–^						
		TLR4^–/–^						
		TLR9^–/–^						
								↑ placental vascular spaces in infected TLR4^–/–^ mice

Rodrigues-Duarte et al.,2018	*P. berghei*	C57BL/6	X	X	X	↓ Stillbirth in TLR4^–/–^ and IFNAR1^–/–^ infected pregnant mice carrying TLR4^+/–^ and IFNAR1^+/–^ progeny	Wildtype and TLR4^–/–^ infected mice with similar litter weight ↑ weight of IFNAR1^+/–^ fetuses from infected IFNAR1^–/–^ mice	X
	NK65	Wildtype						
		TLR4^–/–^						
		IFNAR1^–/–^						

Barboza et al.,2019	*P. berghei*	C57BL/6	X	X	X	X	↓ Fetal weight in MyD88^+/–^ progeny born from MyD88^–/–^	↓ Placental vascular spaces in MyD88^+/–^ placentas from MyD88^–/–^
	NK65	Wildtype						
		MyD88^–/–^						

*The current table content aims to summarize research conducted during the past four decades that was used to inform this review. In summary, *Plasmodium* and murine strains are described for each reference, as well as observations made regarding pregnancy outcomes and placental histology. Information regarding symbology is depicted as follows: Increased (↑), decreased (↓), associated (→), males (♂), females (♀), infected erythrocytes (IEs), hemozoin (Hz), leukocytes (Leu), monocytes/macrophages (MΦ), T cells (Tc), B cells (Bc), NK cells (NKc), dendritic cells (Dc), neutrophils (NΦ), chloroquine (CQ), sulphadoxine-pyrimethamine (SP).*

**TABLE 2:** Compilation of references mentioning the main pathologic manifestations of malaria in pregnancy observed in humans and murine models.

	**Human MiP**	**Murine MiP**
**Pregnancy outcome**		

Recrudescence	Giobbia et al., 2005; Mayor et al., 2009; Laochan et al., 2015; Al Hammadi et al., 2017; Malvy et al.,2018	van Zon and Eling, 1980; Oduola et al., 1982; van Zon et al., 1982, 1985; Marinho et al., 2009; Megnekou et al., 2009; Sharma et al.,2016

Anemia	Brabin et al., 2001; Rogerson et al.,2003	Oduola et al., 1982; Desowitz et al., 1989; Hioki et al., 1990; Megnekou et al., 2009; Poovassery et al., 2009; Avery et al., 2012; Sarr et al., 2012; Sharma et al.,2016

Maternal mortality	Luxemburger et al., 1997; Brabin et al., 2001; Shulman et al., 2002; Nosten et al., 2004; Menéndez et al., 2008; Rogerson et al.,2018	van Zon and Eling, 1980; Oduola et al., 1982; van Zon et al., 1982; Vinayak et al., 1986; Desowitz et al., 1989; Hioki et al., 1990; Pathak et al., 1990; Pavia and Niederbuhl, 1991; Poovassery and Moore, 2006; Neres et al., 2008; Marinho et al., 2009; Megnekou et al., 2009; Poovassery et al., 2009; Rodrigues-Duarte et al., 2012; Sarr et al., 2012; Sharma and Shukla, 2014; Sharma et al.,2016

Stillbirth	Menendez, 1995; Desai et al.,2007	Oduola et al., 1982; Vinayak et al., 1986; Pathak et al., 1990; Rodrigues-Duarte et al., 2012, 2018; Sharma et al.,2016

Abortion	Desai et al.,2007	Poovassery and Moore, 2006, 2009; Neres et al., 2008; Poovassery et al., 2009; Silver et al., 2010; Avery et al., 2012; Sarr et al.,2012, 2015

Preterm delivery	Menendez et al., 2000; Desai et al., 2007; Moore et al.,2017	van Zon et al., 1982; Neres et al.,2008

Reduced newborn/fetal weight	Menendez et al., 2000; Rogerson et al., 2003; Nosten et al., 2004; Desai et al., 2007; Umbers et al.,2011	Oduola et al., 1982; Vinayak et al., 1986; Pathak et al., 1990; Neres et al., 2008; Marinho et al., 2009; Megnekou et al., 2009; Silver et al., 2010; Rodrigues-Duarte et al., 2012, 2018; Conroy et al., 2013; Barboza et al., 2014, 2017, 2019; Sharma and Shukla, 2014; Sharma et al.,2016

Congenital malaria	Rai et al., 2015; Bhatia et al.,2016	Adachi et al.,2000

**Placental histology**		

Parasite accumulation	Walter et al., 1982; Beeson et al., 2002; Beeson and Brown, 2004; Muthusamy et al.,2004, 2007	Oduola et al., 1982, 1986; Vinayak et al., 1986; Desowitz et al., 1989; Pathak et al., 1990; Tegoshi et al., 1992; Poovassery and Moore, 2006, 2009; Neres et al., 2008; Marinho et al., 2009; Megnekou et al., 2009; Rodrigues-Duarte et al., 2012; Sarr et al., 2012; de Moraes et al., 2013; Lima et al., 2014; Sharma and Shukla, 2014; Sharma et al., 2016; Barboza et al.,2017

Hemozoin deposition	Bulmer et al., 1993; Ismail et al.,2000	Oduola et al., 1982, 1986; Vinayak et al., 1986; Neres et al., 2008; Megnekou et al., 2009; Poovassery and Moore,2009

Immune cells infiltrate	Walter et al., 1982; Ordi et al., 1998; Ismail et al., 2000; Rogerson et al., 2007; Othoro et al., 2008; Souza et al.,2013	Oduola et al., 1982, 1986; Tegoshi et al., 1992; Poovassery and Moore, 2006; Neres et al., 2008; Marinho et al., 2009; Poovassery et al., 2009; Barboza et al.,2017

Fibrin Necrosis	Walter et al., 1982; Ismail et al., 2000; Souza et al.,2013	Oduola et al., 1986; Tegoshi et al., 1992; Neres et al., 2008; Poovassery et al., 2009; Avery et al., 2012; Rodrigues-Duarte et al.,2012

Syncytial knots Trophoblast rupture	Ismail et al., 2000; Crocker et al., 2004; Souza et al.,2013	

Trophoblast membrane thickening	Ismail et al., 2000; Souza et al.,2013	Oduola et al., 1982, 1986; Tegoshi et al., 1992; Neres et al., 2008; Marinho et al., 2009; Rodrigues-Duarte et al., 2012; de Moraes et al., 2013; Barboza et al.,2014, 2017, 2019

*Distinct pregnancy outcomes and placental histopathologic alterations are depicted. References used to inform this review are cited in the table according to their contribution in unraveling specific clinical and histologic manifestations of both human and murine malaria in pregnancy.*

## Contribution of Murine Models to the Study of MiP

### Understanding Recrudescence Using Murine Models of MiP

Currently, we have been facing the emerging problem of drug resistance acquisition observed in *P. falciparum* infections, the outcome of which might be the occurrence of recrudescence (without a new infection episode) as a consequence of subcurative therapy (Cattamanchi et al., 2003; Berrevoets et al., 2013). In addition to treatment incapacity to clear circulating parasites, the immune depression observed in specific situations, such as during pregnancy, might elicit the emergence of recrudescent parasites that are either dormant or at submicroscopic levels (Giobbia et al., 2005; Mayor et al., 2009; Laochan et al., 2015; Al Hammadi et al., 2017; Malvy et al., 2018). In this context, some of the first available studies using murine models of MiP appeared during the early 1980s, when van Zon and Eling first described recrudescence in pregnant mice infected with *P. berghei* (van Zon and Eling, 1980; van Zon et al., 1982). Females of different mouse strains (Swiss, C3H/StZ, BALB/c, and B10LP) challenged with *P. berghei* K173 were treated with chloroquine and sulfadiazine to decrease parasitemia and promote the acquisition of immunity before mating. Afterward, pregnancy was shown to induce recrudescence, which was clearly shown to be strain- and gravidity-dependent (van Zon and Eling, 1980) and linked to preterm delivery and maternal mortality (van Zon et al., 1982). Consequently, the pioneering results enabled the authors to draw brief conclusions on similarities observed between human and murine recrudescence by determining that the phenomenon could be associated with (1) the drugs’ incapacity to completely clear the parasite in a previous malaria episode and (2) the particular immune status associated with pregnancy, recapitulating the features of recrudescence in human MiP (Giobbia et al., 2005; Al Hammadi et al., 2017; Malvy et al., 2018). Pregnancy-associated immune modulation was then shown to be linked to increased production of corticoids during murine gestation, thereby facilitating the onset of recrudescence (van Zon et al., 1982, 1985). Notwithstanding the relevance of van Zon and coworkers’ findings, parasite reappearance in maternal circulation was often ensured by experimental reinfection, raising questions about the translational reliability of murine recrudescence results obtained in these models (van Zon and Eling, 1980; van Zon et al., 1982).

Despite the promising advances made during that period, recrudescence in murine models was poorly addressed until the last decade. Only in 2009, two studies have addressed questions about the topic (Marinho et al., 2009; Megnekou et al., 2009). By reevaluating the pioneering experimental model implemented by van Zon and coworkers, the team of Megnekou addressed the production of specific anti-VSA antibodies in pregnant BALB/c mice immunized against *P. berghei* K173. Although previously immunized, protection was lost during pregnancy due to the recrudescence of parasites expressing specific VSA. Consequently, protection was shown to be gravidity-dependent since the less susceptible multiparous mice have raised antibodies against parasite-specific VSA during their previous pregnancies (Megnekou et al., 2009). However, some parasite reappearances were once again ensured by experimental infections, raising the same translational limitations discussed above. During that same year, a study from Marinho et al. (2009) established a model to study recrudescence in BALB/c mice, however, without recurring to mid-gestation reinfection. Accordingly, non-pregnant mice were infected with *P. berghei* ANKA and administered a subcurative treatment to clear apparent parasitemia. Mice were then mated approximately 1 month later and analyzed for recrudescence, which occurred in up to 58% of pregnant mice from gestational day (G)12 onward (Marinho et al., 2009). Maternal mortality and parasitemia were shown to be increased in primigravidae, which diminished with increased gravidity, supporting the observations of Megnekou et al. (2009) regarding acquired immunity in subsequent pregnancies.

Nevertheless, both recrudescent and newly inoculated parasites constitute a risk for highly susceptible pregnant women (Laochan et al., 2015). Accordingly, a recent study has shown that *P. chabaudi* CB sporozoites are capable of inducing recrudescence in pregnant C57BL/6 mice, being more closely related to the human parasite life cycle. However, poor pregnancy outcomes, such as maternal mortality and reduced birth weight, were likely to occur upon heterologous reinfection with *P. chabaudi* AS rather than in the cases of recrudescence. Preacquired immunity against the recrudescent parasite might justify why only reinfection with heterologous parasite lead to poor pregnancy outcomes. Nonetheless, exposure to heterologous *P. chabaudi* AS ensures newborn protection against postnatal infection, reliably resembling the outcomes of human MiP (Sharma et al., 2016).

Although observed in murine MiP, some studies have reported no recrudescence in protocols of treatment and immunization used in mice to control infection before pregnancy (Pathak et al., 1990; Pavia and Niederbuhl, 1991). Explanations might rely on the (1) drug efficiency to clear circulating parasite, (2) usage of less virulent strains (e.g., *P. berghei* NICD and *P. yoelii* 17X), or (3) less susceptible mice strains (e.g., Swiss Albino VRC). Nonetheless, despite some controversial experimental settings, findings associated with the aforementioned murine models have revealed some hidden biological features associated with malaria recrudescence during pregnancy that otherwise would be impossible to address.

### Understanding Poor Pregnancy Outcomes Using Murine Models of MiP

A multitude of outcomes can occur as a result of developing MiP. When considering the areas of *P. falciparum* frequent and stable transmission, pregnancy-associated consequences might depend on several aspects such as infection trimester, gravidity and maternal health status that ultimately dictate the onset of maternal anemia, fetal and maternal mortality, abortion, and reduced birth weight as consequences of IUGR and/or preterm delivery (Desai et al., 2007; Umbers et al., 2011; Rogerson et al., 2018). Outcomes in human pregnancy are accessible for study; however, in addition to ethical constraints, illogical and incorrect correlations might be drawn from imprecise information given by patients enrolling in these prospective studies, such as imprecise time of infection or clinical status. Fittingly, murine models provided the opportunity to investigate MiP outcomes in a controlled experimental setting.

#### Maternal Anemia

Maternal anemia that develops during MiP is considered a significant risk factor for poor pregnancy outcomes and is closely associated with increased parasite burden and reduced birth weight (Rogerson et al., 2003). Accordingly, several distinct experimental murine models accurately recapitulate anemia observed during human MiP. Similarly, *P. chabaudi* AS infection potentiates the onset of anemia (reduced hematocrit percentage) in pregnant C57BL/6 mice. Anemia was therefore linked to a rapid increase in parasitemia, which peaks abruptly in pregnant mice (Poovassery and Moore, 2006; Poovassery et al., 2009; Avery et al., 2012; Sarr et al., 2012). This phenomenon was also shown to occur in different murine strains, such as BALB/c mice (Hioki et al., 1990) and Wistar rats (Desowitz et al., 1989), infected with *P. berghei* NK65 and NYU-2, respectively, in which hemoglobin and hematocrit decreased as a result of increased parasitemia. Reproducibility of anemia is expected among different experimental models since hemolysis constitutes an intrinsic step in the *Plasmodium* spp. life cycle in both rodents and humans. Nevertheless, this critical parameter has been poorly investigated in murine malaria, in which its relationship with infection timing, gravidity, and pregnancy outcomes were never addressed as in humans (Brabin et al., 2001; Rogerson et al., 2003).

#### Maternal Mortality

Maternal mortality seems to be a less frequent outcome in pregnancies complicated by malaria (Menéndez et al., 2008; Rogerson et al., 2018). Maternal death associated with *Plasmodium* spp. infection tends to occur more frequently in areas of low malaria transmission due to the reduced level of premunition (Nosten et al., 2004). Nevertheless, pregnant women with malaria who live in endemic areas are also prone to die due to severe anemia (Brabin et al., 2001; Shulman et al., 2002). Likewise, both pregnant mice and rats infected with murine *Plasmodium* spp. strains were shown to die during gestation, which was dependent on the parasite strain, infection timing, and host intrinsic characteristics. Regarding parasite strains, it was demonstrated that *P. berghei* (van Zon and Eling, 1980; Oduola et al., 1982; van Zon et al., 1982; Vinayak et al., 1986; Desowitz et al., 1989; Hioki et al., 1990; Pathak et al., 1990; Neres et al., 2008; Marinho et al., 2009; Megnekou et al., 2009; Rodrigues-Duarte et al., 2012; Sharma and Shukla, 2014), *P. yoelii* (Pavia and Niederbuhl, 1991), and *P. chabaudi* (Poovassery and Moore, 2006, 2009; Poovassery et al., 2009; Sarr et al., 2012; Sharma et al., 2016) induce maternal death in a considerable variety of experimental settings. However, only one study has directly compared this outcome induced by different parasites, clearly showing increased mortality rates in pregnant C57BL/6 mice infected with *P. berghei* NK65 compared to K173 and ANKAΔpm4 strains (Rodrigues-Duarte et al., 2012). One must consider that mortality rates also depend on host susceptibility to infection since, for instance, pregnant A/J mice experience a higher risk of mortality than C57BL/6 mice when infected with *P. chabaudi* AS (Sarr et al., 2012). These findings support the notion that maternal survival is dependent on both parasite and host intrinsic features. Similarly, mortality was shown to increase in pregnant C57BL/6 and BALB/c mice infected with *P. chabaudi* (Poovassery and Moore, 2006; Poovassery et al., 2009) or *P. berghei* (Neres et al., 2008; Rodrigues-Duarte et al., 2012), respectively, when compared to their non-pregnant counterparts. This is a well-known phenomenon that also occurs in humans who are primarily in areas of unstable malaria transmission (Luxemburger et al., 1997; Nosten et al., 2004). Infection timing was also shown to be linked to maternal mortality, which primarily increases in cases of early gestation infection. This was demonstrated to occur in Wistar rats (Desowitz et al., 1989), as well as in Swiss Albino, A/J, ICR, and BALB/c mice (Oduola et al., 1982; Vinayak et al., 1986; Hioki et al., 1990) infected with *P. berghei*. However, mid-gestation infection was shown to lead to increased survival with a higher number of animals achieving parturition. Interestingly, it is unclear whether this occurs in humans and if first trimester infections lead to increased maternal mortality rates (Desai et al., 2007; Rogerson et al., 2018). Nevertheless, regardless of the infection trimester, mortality is highly dependent on gravidity and is more prevalent in primigravidae than in multiparous women (Menéndez et al., 2008). Equally, mice tend to experience the same effect, which is likely to be dependent on immunity acquired during previous gestations (van Zon and Eling, 1980; Marinho et al., 2009). Accordingly, immunity seems to be pivotal in reduced murine mortality, as ICR mice immunized with attenuated *P. yoelii* before pregnancy had improved survival rates when challenged with the lethal *P. yoelii* 17X strain (Pavia and Niederbuhl, 1991). Although a direct causality has never been proved, increased mortality observed in murine models of MiP seem to be associated with the development of systemic infection measured by increased peripheral parasitemia that correlates with decreased body weight and patent patterns of anemia (Poovassery et al., 2009; Sarr et al., 2012).

Considering the discussion above, it is clear that mortality during pregnancy depends on a wide range of factors that ultimately will affect host survival. Most of them are commonly recapitulated in rodent experimental models in which experiments have contributed with important findings in this regard. However, one must carefully consider the often-fatal outcomes of murine infections induced by highly lethal parasites that are dissimilar to the infrequent lethality observed in human MiP.

#### *In uterus* Death and Abortion

*In uterus* death and abortion are rather infrequent outcomes of pregnancies complicated by malaria. However, there is a clear association between MiP development and stillbirth incidence, which is noted as being higher in women with perceivable placental parasitemia (Desai et al., 2007). In contrast, the association between abortion and MiP seems to be rarely discussed, with only scarce reports of this event occurring in low transmission areas, probably resulting from early trimester infections (Menendez, 1995; Desai et al., 2007). It is important to highlight that logistical constraints imposed by cultural and socioeconomic boundaries are the reason by which it is difficult to assess this particular pregnancy outcome. Interestingly, the incidence of abortion seems to be much more frequent in murine models than in humans. Accordingly, *P. chabaudi* AS infection of pregnant C57BL/6 mice revealed a rather frequent pattern of abortion and reduced fetal viability at mid-gestation (G10-12) when the parasite was inoculated immediately after conception. This event overlaps with the peripheral parasitemia peak and massive accumulation of parasites inside the placenta (Poovassery and Moore, 2006). As expected, the parasitemia peak and abortion were frequently observed in mice having increased production of cytokines in both peripheral blood (e.g., IL-1β and IL-10) and placenta (e.g., IFN-γ) (Poovassery and Moore, 2009). In a follow-up study, it was shown that fetal loss and reduced viability in pregnant C57BL/6 mice infected with *P. chabaudi* AS were markedly influenced by IFN-γ and TNF-α production (Poovassery et al., 2009) and increased coagulopathy and impaired fibrinolysis (Avery et al., 2012). In the same extent, abortion was shown to occur concomitantly with apoptosis of inflammatory cells and spongiotrophoblast in the junctional zone (between the uterus and the labyrinth), which has been discussed to occur in a way that is dependent on TNF-α signaling (Sarr et al., 2015). Altogether, these findings established a clear link between the immune response triggered against malaria and poor pregnancy outcomes. Moreover, models of abortion are also characterized by early infection with *P. chabaudi*, but stillbirth has also been observed in Swiss Albino (Vinayak et al., 1986; Pathak et al., 1990), BALB/c (Neres et al., 2008), and C57BL/6 (Rodrigues-Duarte et al., 2012) mice infected with *P. berghei* at mid-gestation. In these experimental models, reduced fetal viability without clear abortion observed at late gestation somewhat reflects stillbirth occurring in humans during the third gestational trimester (Desai et al., 2007).

Notably, it is important to highlight that translating these findings to human research may raise some serious controversies. Human stillbirth is often described as a dead conceptus that was expelled or removed from the womb 22 weeks after conception, being no longer considered an abortion (less than 22 weeks) (Lawn et al., 2016). In contrast, in the aforementioned murine models, there is no parallel measure at which fetal viability was assessed that considers gestational age. Vaginal secretions containing blood are normally taken as an indicative of early abortion, followed by the observation of necrotic structures (resorptions) that lack fetal or placental morphology (Poovassery and Moore, 2006; Neres et al., 2008). On the other hand, fetal viability is normally evaluated closer to term, after touching *in uterus* or delivered conceptus with pliers. Fetuses that fail to react are considered stillbirths (Neres et al., 2008). Therefore, definitions in these situations are often misleading, and conclusions should be made with extreme caution since abortion and stillbirth etiology may vary between rodent and human MiP.

#### Preterm Birth

Preterm birth, which encompasses every live birth that occurs before the 37th week of gestation (Goldenberg et al., 2009), is a more frequent consequence and is one of the leading causes of reduced birth weight associated with MiP (Menendez et al., 2000; Desai et al., 2007; Moore et al., 2017). Interestingly, the occurrence of spontaneous delivery in mice or rats in the context of MiP is either poorly addressed or considerably infrequent. One study reported the occurrence of preterm delivery of dead pups from pregnant BALB/c mice infected with *P. berghei* ANKA (Neres et al., 2008). However, this definition does not seem to fit the circumstance since preterm birth assumes that the progeny is born alive before term, which is unlikely to occur before G19 (reviewed elsewhere McCarthy et al., 2018). To the same extent, murine models have been questioned regarding their capacity to reproduce human preterm birth due to inaccurate measurements and non-standardized markers.

In this regard, evaluation of preterm birth in murine models of MiP is probably far from replicating human pathology. However, a better evaluation of this outcome may lead to improved disease models with more reliable translation capacities (McCarthy et al., 2018).

#### Progeny Outcomes and Development

Progeny outcomes and development will ultimately be severely affected as a result of the aforementioned clinical and obstetric manifestations during MiP. Often resulting from IUGR and/or preterm delivery, reduced birth weight contributes significantly to postnatal mortality and impaired child development across the malaria endemicity spectrum and is clearly more frequent in women during their first pregnancies (Nosten et al., 2004; Desai et al., 2007). It is unclear how MiP truly leads to growth restriction, yet evidences point to a multitude of factors such as angiogenic imbalance, endocrine dysregulation, deficiencies in transplacental nutrient transportation, severe inflammation and placental insufficiency (Umbers et al., 2011). During the 1980s, some studies reported the first observations of altered birth weight in mice suffering from MiP, which was found to be significantly diminished in pups from A/J, ICR (Oduola et al., 1982) and Swiss albino (Vinayak et al., 1986) litters born from mice infected with *P. berghei* NK65 and NICD, respectively. It seems that impaired progeny development is a rather well-conserved outcome of murine MiP since it was reported in BALB/c (Neres et al., 2008; Marinho et al., 2009; Megnekou et al., 2009; Silver et al., 2010) and C57BL/6 (Rodrigues-Duarte et al., 2012; Sharma et al., 2016) mice infected with a wide variety of parasite strains, suggesting the existence of conserved pathogenesis mechanisms (Rodrigues-Duarte et al., 2012). This pathologic outcome seems to be avoided with on-time administration of antimalarial drugs such as chloroquine and sulphadoxine-pyrimethamine, which were shown to abrogate systemic parasitemia, therefore improving offspring weight at delivery (Sharma and Shukla, 2014). Additionally, as it occurs in humans, progeny birth weight improved in mouse multigravidae. This trait was clearly shown to be gravidity-dependent, as pups belonging to the first litter were much smaller on average than those born from subsequent pregnancies when the corresponding dams were induced with MiP by recrudescent *P. berghei* ANKA (Marinho et al., 2009).

Further research conducted in the last decade revealed some hidden aspects of reduced birth weight etiology that would not be revealed without the wide range of genetically manipulated mice strains. Strikingly, it seems that proper fetal development is impaired upon the activation of specific components linked to innate immunity, such as complement system receptor (C5aR), Toll-like receptor 4 (TLR4), type I interferon receptor 1 (IFNAR1), and adaptor protein myeloid differentiation factor 88 (MyD88). BALB/c C5aR^–/–^ (Conroy et al., 2013), C57BL/6 TLR4^–/–^ (Barboza et al., 2017; Rodrigues-Duarte et al., 2018), C57BL/6 IFNAR^–/–^ (Rodrigues-Duarte et al., 2018), and C57BL/6 MyD88^–/–^ (Barboza et al., 2014, 2019) mice were shown to have litters with normal fetal weight, development and survival, even though being infected with *P. berghei* ANKA (in BALB/c) or *P. berghei* NK65 (in C57BL/6). These findings substantially advanced the field by strikingly implying the innate immune system on MiP-associated reduced birth weight pathogenesis, enabling future preclinical trials of anti-inflammatory drugs (e.g., the TLR4 antagonist IAXO) (Barboza et al., 2017) to be used concomitantly with antimalarial therapies to prevent MiP-associated poor pregnancy outcomes. However, progress in this field should also be done with extreme caution due to possible undesired side effects resulting from adjunctive therapies to treat severe malaria such as those observed in trials conducted with anti-TNF-α therapy to treat cerebral malaria and others (van Hensbroek et al., 1996; Varo et al., 2018).

Moreover, the postnatal scenario of human MiP might rarely include the appearance of congenital malaria, whose onset is tightly controlled by transplacental transmission of maternal antibodies to the fetus, resulting in isolated and scarce events often diagnosed as neonatal sepsis (Rai et al., 2015; Bhatia et al., 2016). Conceptus infection might occur due to blood exchanges at delivery or during pregnancy due to loss of villi integrity and syncytium rupture (Crocker et al., 2004; Robbins and Bakardjiev, 2012; Souza et al., 2013). By contrast, the phenomenon is thought to be even rarer in rodents. In related studies, the authors have clearly stated that no congenital malaria was observed, despite an increased fetal spleen weight, which might an indicative of fetal infection (Oduola et al., 1982; Vinayak et al., 1986). However, work by Adachi et al. (2000) addressed this question under the assumption that both mice and rats rarely transmit the parasite to their offspring. Accordingly, the authors detected parasites by nested PCR in a few pups born from pregnant BALB/c mice infected with *P. berghei*. These findings support the rareness of the event due to the lack of a uniform presence of parasites, even in pups within the same litter. The hemotrichorial layer in the murine placenta presents a much thicker barrier against transplacental passage of parasites than the human hemomonochorial placenta (Crocker et al., 2004), which might explain the reduced incidence of murine congenital malaria. Nevertheless, discrepancies in existing reports contraindicate the usage of murine models in this regard, identifying them as unsuitable for studying this rare disease outcome.

In conclusion, one may face challenges when translating pregnancy outcome findings obtained in murine models to human pathology. Nevertheless, significant achievements have been made in recent years, despite the considerable diversity of experimental settings and non-standardized methodologies. Improving them will certainly lead to enriched results and more accurate and meaningful conclusions taken from murine models of MiP.

### Understanding Placental Pathology Using Murine Models of MiP

Most of the aforementioned outcomes occurring in pregnancies complicated by malaria are strongly associated with the dysregulation of placental homeostasis due to a significant accumulation of parasite-infected erythrocytes inside this organ, which overlaps with the onset of PM (Sharma and Shukla, 2017). Accordingly, several cytological and histological abnormalities are easily observed in infected placentas in addition to parasite accumulation, such as the malarial pigment hemozoin, immune cells, syncytial knots, fibrin deposition, necrosis and placental barrier thickening (Walter et al., 1982; Ismail et al., 2000; Souza et al., 2013). Although despite being PM hallmarks, not all of these histopathologic features are accurately shown by murine models of MiP (schematically represented in Figure 3).

#### Parasitized Erythrocytes

Parasitized erythrocytes tend to selectively accumulate in the placenta (Figures 1C,D), achieving greater parasitemia levels than those observed in the peripheral blood of pregnant infected women (Beeson et al., 2002; Muthusamy et al., 2004). It is unquestionable that murine parasites also accumulate inside the placenta (Figure 2D) since a large number of studies of placental histopathology noted this finding for a wide range of strains (Table 1). Notably, Poovassery and Moore (2006) have demonstrated that *P. chabaudi* AS parasites also accumulate preferentially inside the placenta of C57BL/6 mice, leading to greater placental parasitemia than that observed in peripheral blood. Interestingly, these murine parasite populations were shown to be mainly constituted by mature forms with an almost null percentage of early ring-stage parasites (Megnekou et al., 2009), which is in line with observations made during *P. falciparum* infections (Beeson et al., 2002). Similarly, intravital microscopy studies performed in placentas from pregnant mice infected either with *P. berghei* or *P. chabaudi* also suggested that those that accumulate are indeed the mature forms of the parasite (Lima et al., 2014), which preferably adhere to the trophoblast in areas of low blood flow inside the maternal blood sinusoids (de Moraes et al., 2013). As observed for *P. falciparum* regarding its preferential adhesion to CSA and controversial binding to hyaluronic acid (HA) (Beeson and Brown, 2004; Muthusamy et al., 2007), the adhesion of *P. berghei* was also shown to be dependent on placental CSA and HA as demonstrated by adhesion assays of mice IEs to placental sections treated with chondroitinase or hyaluronidase (Neres et al., 2008; Marinho et al., 2009). These findings once again support similar pathogenesis mechanisms between human and murine PM despite the lack of known PfEMP1 homologs (Hviid et al., 2010).

#### The Malarial Pigment Hemozoin

The malarial pigment hemozoin, a byproduct of hemoglobin catabolism performed by *Plasmodium* spp. to detoxify free heme (Olivier et al., 2014), is frequently observed in placentas from infected pregnant women (Figures 1E,F) and is often used as an indicator of PM, even in the absence of detectable parasites (Bulmer et al., 1993; Ismail et al., 2000). Similarly, placental infection by murine parasites also leads to hemozoin deposition (Figure 2F). Oduola and coworkers first observed this phenomenon in histologic sections of placentas from different murine backgrounds infected with *P. berghei* NK65. Shortly thereafter, the pigment was exclusively observed in maternal blood sinusoids, whose concentrations increased with augmented parasite loads. Accordingly, hemozoin-containing monocytes were also frequently observed (Oduola et al., 1982, 1986). In addition, hemozoin was also detected in trophoblast giant cells, suggesting an active phagocytic process (Poovassery and Moore, 2009). The pigment is frequently detected under polarized light microscopy (Neres et al., 2008; Megnekou et al., 2009) and is used to indicate past-chronic PM in humans according to the diagnostic criteria of Bulmer et al. (1993). To our knowledge, this event was rarely observed in murine models (Oduola et al., 1986). The fact that hemozoin was rarely observed alone in infected placentas suggests that the experimental settings from current models fail to reproduce past-chronic infections and are only able to reproduce acute PM.

#### Immune Cell Infiltrate

Immune cell infiltrate, which occurs as a response to parasite/hemozoin accumulation inside the placenta (Figure 1D), is one of the key hallmarks of the disease and is often associated with poor pregnancy outcomes (Menendez et al., 2000; Rogerson et al., 2003; Umbers et al., 2011). Monocytes/macrophages are the most abundant constituents of these inflammatory infiltrates, and recruitment is widely correlated with placental production of MIP-1α, MCP-1, I-309, and IL-8 chemokines (Abrams et al., 2003). Not surprisingly, the same event was observed in placentas from mice (Figure 2E) and rats infected with *P. berghei*, especially in those containing detectable placental parasites (Oduola et al., 1982, 1986; Tegoshi et al., 1992). Notably, the infiltrate, which was mainly composed of monocytes (CD11b^+ve^ cells detected by cytochemistry) and macrophages (CD11b^+ve^ cells detected by cytochemistry and *Cd68*- and *Mgl2*-expressing cells identified by qPCR), was linked to the production of some attractant chemokines, such as MIP-1α and MCP-1 (Neres et al., 2008; Marinho et al., 2009; Sarr et al., 2015; Barboza et al., 2017). However, the event is far from reflecting chronic intervillositis observed in humans (Ordi et al., 1998), probably due to the shorter gestational period in rodents. Additionally, some authors have commented on the scarcity of this event, especially in placentas from C57BL/6 mice infected with *P. chabaudi*, which tend to have fewer accumulated monocytes/macrophages due to reduced placental parasite burden (Poovassery and Moore, 2006; Poovassery et al., 2009). Early reports on different leukocyte populations in infected murine placentas noted the existence of mononuclear (Tegoshi et al., 1992) and polymorphonuclear (Oduola et al., 1986) cells. Molecular biology methods were later used to dissect these cell types, dividing them into dendritic cells (*Mgl2*), neutrophils (*Ncf2*), NK cells (*Klrd1*), T (*Cd3e*), and B (*Cd22*) lymphocytes (gene expression quantification by qPCR) (Marinho et al., 2009; Sarr et al., 2015; Barboza et al., 2017). Remarkably, this is in line with observations performed in human PM regarding the placental accumulation of NK cells, T lymphocytes and other non-specified polymorphonuclear cells (Ordi et al., 2001; Rogerson et al., 2007; Othoro et al., 2008). Nevertheless, despite the clear accumulation of immune cells in murine placentas, its association with poor pregnancy outcomes remains to be elucidated in experimental models that oppose current knowledge of human pathology (Rogerson et al., 2003; Umbers et al., 2011).

#### Placental Fibrinoid Necrosis

Placental fibrinoid necrosis normally occurs as a consequence of extensive placental tissue damage caused during *Plasmodium* spp. infection (Figure 1B). In fact, fibrin deposition is initiated to promote placental tissue repair but soon becomes cytotoxic, leading to necrosis (Walter et al., 1982; Ismail et al., 2000) and poor pregnancy outcomes such as premature delivery and reduced birth weight (Menendez et al., 2000; Avery et al., 2012). Likewise, pioneering studies from the 1980s revealed the presence of fibrinoid necrosis in placental sections from mice and rats infected with *P. berghei*, although without clear conclusions on its consequences (Oduola et al., 1986; Tegoshi et al., 1992). Later, fibrinoid necrosis was reported in placentas from *P. berghei*-infected BALB/c mice (Figure 2B), which delivered litters with a patently reduced birth weight phenotype (Neres et al., 2008), and fibrin thrombi in placentas from C57BL/6 mice infected with *P. chabaudi* that experienced spontaneous abortion (Poovassery et al., 2009). Discussion was taken to the point in which fibrin deposition in maternal blood sinusoids would significantly impair placental capability to perform physiological tasks such as respiration and nutrient exchanges, probably due to clotting, blood arrest, necrosis and trophoblast death. In a subsequent study, Avery et al. (2012) showed by western blot that fibrin deposits were increased in placentas from C57BL/6 mice infected with *P. chabaudi*, which occurred in parallel with an upregulation of coagulation-associated genes. These findings established an important association between impaired fibrinolysis and coagulation and the poor pregnancy outcomes of MiP.

#### Labyrinth Disarrangement

Labyrinth disarrangement will ultimately reflect murine placental dysfunction, which is characterized by particular histological alterations that have considerable differences from human PM, mostly due to the existence of a widely different villi structure (Figures 1A,B vs. Figures 2A–D). Of note, two features of human PM that have no counterparts in infected murine placentas are syncytiotrophoblast rupture (previously discussed in the context of congenital malaria) and syncytial knots (Ismail et al., 2000; Souza et al., 2013). These protrusions of syncytial nuclear aggregates, which have been associated with hypoxia and oxidative stress in human placentas (Heazell et al., 2007), were discussed as having no similar structure in murine placentas that could be detected under light or electron microscopy (Tegoshi et al., 1992). Syncytial knots, which are considered an accumulation of degrading nuclei, was once erroneously described as a phenomenon of trophoblast hyperplasia [discussed elsewhere (Heazell et al., 2007)]. To our knowledge, the latter was never clearly discussed in the context of human PM. Accordingly, there are unclear reports of trophoblast hyperplasia occurring in the placentas of mice (Oduola et al., 1986; Vinayak et al., 1986; Pathak et al., 1990; Neres et al., 2008) and rats (Oduola et al., 1986; Tegoshi et al., 1992) infected with *P. berghei*. Nevertheless, evidence of this event includes unclear microscopy images that fail to address the apparent enlargement/swelling of tissue that occurs due to cell proliferation. However, there is a striking thickening of the trophoblast basal membrane that partially overlaps with the concept of tissue swelling. In human PM, this frequent phenomenon can be qualitatively analyzed (Ismail et al., 2000) or more accurately quantified as the distance that separates fetal capillaries from villi outer membrane (Souza et al., 2013). Regardless of the methodology used, this parameter was found to be significantly thicker during human PM and, being discussed as strongly influencing transplacental transport of vital compounds. Similarly, this was qualitatively analyzed and reported in mouse and rat placentas infected with *P. berghei*, which was hypothesized to be a consequence of the fibrotic process resulting from massive tissue repair (Oduola et al., 1986; Tegoshi et al., 1992). Later, some other works have developed methods to indirectly quantify trophoblast thickening, taking advantage of the sinusoidal nature of mouse placentas. Accordingly, maternal blood areas were quantified, and the reduction of vascular spaces in placentas from BALB/c mice infected with *P. berghei* ANKA was considered a proxy for basal membrane thickening (Neres et al., 2008). This supports the conclusion that reduced maternal blood spaces due to trophoblast membrane thickening and maternal sinusoidal remodeling (de Moraes et al., 2013) would ultimately lead to placental insufficiency and impaired transplacental transport of nutrients [discussed elsewhere (Neres et al., 2008; de Moraes et al., 2013)]. The same methodology was further used to determine that distinct parasites inflict different magnitudes of circulatory impairment and membrane thickening in placentas from C57BL/6 mice (Rodrigues-Duarte et al., 2012). The etiology of the event was further addressed in *P. berghei* NK65-infected C57BL/6 TLR4^–/–^ and MyD88^–/–^ KO mice, which had blood sinusoidal areas similar to those observed in non-infected pregnant mice (Barboza et al., 2014, 2017). These findings established a logical link between host innate immunity and placental pathology, once again supporting the notion that outcomes of MiP mostly result from damage and homeostatic dysregulation inflicted mostly by factors of an immunologic nature.

## Conclusion

Clearly, the development of murine models that recapitulate traits from human MiP has definitively contributed to the current understanding of this disease. However, there are still few reports that truly reveal some of the pathogenesis mechanisms of MiP. Only in the last decade have some studies clearly unraveled some hidden molecular mechanisms of MiP, such as innate immunity activation and its contributions to poor pregnancy outcomes (Poovassery et al., 2009; Conroy et al., 2013; Barboza et al., 2014, 2017, 2019; Rodrigues-Duarte et al., 2018). Indeed, the advent of genetic engineering and the capacity to generate a wide range of knockout mice were definite turning points from which we are still benefiting. Until this point, most studies have tried to establish proper experimental settings that would ultimately validate murine models as suitable for studying MiP. First, striking similarities are observed regarding evolutionary and developmental traits between murine and human placentas, from which one might conclude that physiologically, both would behave in a particularly similar way (Georgiades et al., 2002; Wildman et al., 2006). Additionally, murine parasites exhibit a group of characteristics that support the conclusion that murine pathology would somewhat resemble human MiP, despite the well-known differences between rodent *Plasmodium* species and *P. falciparum* biology (Lamb et al., 2006; Hviid et al., 2010). Nevertheless, one might consider the patent limitations of conclusions regarding disease outcomes, which can later limit the translational capacities of observed results. As such, refinement of experimental design and standardization of methodology is necessary for the improvement of such models (BOX 1). Resolving these gaps will certainly enrich research in the field, possibly reaffirming the usage of murine models to address more specific and complex questions implicit in drug preclinical trials and vaccine development (Doritchamou et al., 2017). Research in the field has indeed overcome several milestones due to the usage of rodent models with much progress that is still to come.

**Box 1. Suggestions to improve future murine models of MiP.**

Evolution has grouped murine animals and humans in similar phylogenetic clades according to some striking similarities. However, it has also separated them especially from an anatomically perspective. Some differences cannot be surpassed; yet, our understanding of some diseases and complex biological processes can be improved with the refinement of experimental design and standardization of analysis methods when using animal models such as experimental murine models of MiP. As such, our understanding of MiP could be improved by addressing some of the following points:

• Understanding the adhesion mechanism of murine parasite strains.
• Identification of VAR2CSA functional homologs in murine parasites.
• Standardization of protocols using specific species of murine parasites to differently address specific aspects of uncomplicated or severe MiP (e.g., usage of less or more virulent parasite strains, tropism for reticulocytes or mature erythrocytes, preferential tissue for accumulation).
• Standardized definitions and accurate measures for abortion, preterm delivery, stillbirth and placental malaria in the context of murine MiP.
• Investigating the possibility of congenital malaria in murine models of MiP.

## Author Contributions

AB drafted the manuscript, compiled information from the literature, and designed the figures and tables. MP drafted the manuscript and gathered information from the literature. SE supervised and reviewed the manuscript. CM supervised and reviewed the manuscript and designed the figures and tables.

## Conflict of Interest Statement

The authors declare that the research was conducted in the absence of any commercial or financial relationships that could be construed as a potential conflict of interest.

## References

[B1] Abrams E. T. Brown H. Chensue S. W. Turner G. D. H. Molyneux M. E. Meshnick S. R. (2003). Host response to malaria during pregnancy: placental monocyte recruitment is associated with elevated β chemokine expression. *J. Immunol.* 170 2759–2764. 10.4049/jimmunol.170.5.2759 12594307

[B2] Adachi M. Yuda M. Ando K. Sakurai M. Chinzei Y. (2000). Scant parasitemia in BALB/c mice with congenital malaria infection. *J. Parasitol.* 86 1030–1034. 10.1645/0022-3395(2000)086 11128475

[B3] Al Hammadi A. Mitchell M. Abraham G. M. Wang J. P. (2017). Recrudescence of *Plasmodium falciparum* in a primigravida after nearly 3 years of latency. *Am. J. Trop. Med. Hyg.* 96 642–644. 10.4269/ajtmh.16-0803 28044045 PMC5361538

[B4] Avery J. W. Smith G. M. Owino S. O. Sarr D. Nagy T. Mwalimu S. (2012). Maternal malaria induces a procoagulant and antifibrinolytic state that is embryotoxic but responsive to anticoagulant therapy. *PLoS One* 7:e31090. 10.1371/journal.pone.0031090 22347435 PMC3274552

[B5] Barboza R. Hasenkamp L. Barateiro A. Murillo O. Peixoto E. P. M. Lima F. A. (2019). Fetal-derived MyD88 signaling contributes to poor pregnancy outcomes during gestational malaria. *Front. Microbiol.* 10:68. 10.3389/fmicb.2019.00068 30761111 PMC6362412

[B6] Barboza R. Lima F. A. Reis A. S. Murillo O. J. Peixoto E. P. M. Bandeira C. L. (2017). TLR4-mediated placental pathology and pregnancy outcome in experimental malaria. *Sci. Rep.* 7 1–12. 10.1038/s41598-017-08299-x 28819109 PMC5561130

[B7] Barboza R. Reis A. S. Silva L. G. Da Hasenkamp L. Pereira K. R. B. Câmara N. O. S. (2014). MyD88 signaling is directly involved in the development of murine placental malaria. *Infect. Immun.* 82 830–838. 10.1128/IAI.01288-1213 24478096 PMC3911391

[B8] Beeson J. G. Amin N. Kanjala M. Rogerson S. J. (2002). Selective accumulation of mature asexual stages of plasmodium falciparum -infected erythrocytes in the placenta. *Infect. Immun.* 70 5412–5415. 10.1128/IAI.70.10.5412 12228265 PMC128361

[B9] Beeson J. G. Brown G. V. (2004). Plasmodium falciparum–infected erythrocytes demonstrate dual specificity for adhesion to hyaluronic acid and chondroitin sulfate a and have distinct adhesive properties. *J. Infect. Dis.* 189 169–179. 10.1086/380975 14722880

[B10] Berrevoets M. A. H. Sprong T. Meis J. F. Dofferhoff A. S. M. (2013). Plasmodium falciparum malaria recrudescence occurring 2.5 years after leaving an endemic country. *Neth. J. Med.* 71 426–428. 24127503

[B11] Bhatia R. Rajwaniya D. Agrawal P. (2016). Congenital malaria due to plasmodium vivax infection in a neonate. *Case Rep. Pediatr.* 2016 1–2. 10.1155/2016/1929046 27651968 PMC5019906

[B12] Brabin B. J. Hakimi M. Pelletier D. (2001). An analysis of anemia and pregnancy-related maternal mortality. *J. Nutr.* 131 604S–615S. 10.1093/jn/131.2.697S 11160593

[B13] Bulmer J. N. Rasheed F. N. Francis N. Morrison L. Greenwood B. M. (1993). Placental malaria.1. Pathological Classification. Histopathology 22 211–218. 8495954 10.1111/j.1365-2559.1993.tb00110.x

[B14] Burton G. J. Hempstock J. Jauniaux E. (2001). Nutrition of the human fetus during the first trimester - a review. *Placenta* 22 70–76. 10.1053/plac.2001.0639 11312634

[B15] Burton G. J. Watson A. L. Hempstock J. Skepper J. N. Jauniaux E. (2002). Uterine glands provide histiotrophic nutrition for the human fetus during the first trimester of pregnancy. *J. Clin. Endocrinol. Metab.* 87 2954–2959. 10.1210/jcem.87.6.8563 12050279

[B16] Cattamanchi A. Kyabayinze D. Hubbard A. Rosenthal P. J. Dorsey G. (2003). Distinguishing recrudescence from reinfection in a longitudinal antimalarial drug efficacy study: comparison of results based on genotyping of MSP-1. MSP-2, and GLURP. *Am. J. Trop. Med. Hyg.* 68 133–139. 10.1186/1475-2875-5-127 12641400

[B17] Conroy A. L. Silver K. L. Zhong K. Rennie M. Ward P. Sarma J. V. (2013). Complement activation and the resulting placental vascular insufficiency drives fetal growth restriction associated with placental malaria. *Cell Host Microbe* 13 215–226. 10.1016/j.chom.2013.01.010 23414761

[B18] Crocker I. P. Walraven G. Tanner O. M. Baker P. N. Myers J. E. Bulmer J. N. (2004). Syncytiotrophoblast degradation and the pathophysiology of the malaria-infected placenta. *Placenta* 25 273–282. 10.1016/j.placenta.2003.09.010 15028419

[B19] de Moraes L. V. Tadokoro C. E. Gómez-Conde I. Olivieri D. N. Penha-Gonçalves C. (2013). Intravital placenta imaging reveals microcirculatory dynamics impact on sequestration and phagocytosis of plasmodium-infected erythrocytes. *PLoS Pathog.* 9:e1003154. 10.1371/journal.ppat.1003154 23382682 PMC3561179

[B20] Dellicour S. Tatem A. J. Guerra C. A. Snow R. W. Ter Kuile F. O. (2010). Quantifying the number of pregnancies at risk of malaria in 2007: a demographic study. *PLoS Med.* 7:e1000221. 10.1371/journal.pmed.1000221 20126256 PMC2811150

[B21] Desai M. ter Kuile F. O. Nosten F. McGready R. Asamoa K. Brabin B. (2007). Epidemiology and burden of malaria in pregnancy. *Lancet Infect. Dis.* 7 93–104. 10.1016/S1473-3099(07)70021-X 17251080

[B22] Desowitz R. S. Shida K. K. Pang L. Buchbinder G. (1989). Characterization of a model of malaria in the pregnant host: plasmodium berghei in the white rat. *Am. J. Trop. Med. Hyg.* 41 630–634. 10.4269/ajtmh.1989.41.630 2701632

[B23] Doritchamou J. Teo A. Fried M. Duffy P. E. (2017). Malaria in pregnancy: the relevance of animal models for vaccine development. *Lab Anim.* 46 388–398. 10.1038/laban.1349 28984865 PMC6771290

[B24] Duffy P. E. Fried M. (2001). “Malaria in pregnancy,” in *Deadly Parasite, Susceptible Host*, 1st Edn, eds Duffy P. E. Fried M. (London: Taylor & Francis).

[B25] Franke-Fayard B. Fonager J. Braks A. Khan S. M. Janse C. J. (2010). Sequestration and tissue accumulation of human malaria parasites: can we learn anything from rodent models of malaria? *PLoS Pathog.* 6:e1001032. 10.1371/journal.ppat.1001032 20941396 PMC2947991

[B26] Fried M. Domingo G. J. Gowda C. D. Mutabingwa T. K. Duffy P. E. (2006). Plasmodium falciparum: chondroitin sulfate A is the major receptor for adhesion of parasitized erythrocytes in the placenta. *Exp. Parasitol.* 113 36–42. 10.1016/j.exppara.2005.12.003 16430888

[B27] Georgiades P. Fergyson-Smith A. C. Burton G. J. (2002). Comparative developmental anatomy of the murine and human definitive placentae. *Placenta* 23 3–19. 10.1053/plac.2001.0738 11869088

[B28] Giobbia M. Tonon E. Zanatta A. Cesaris L. Vaglia A. Bisoffi Z. (2005). Late recrudescence of Plasmodium falciparum malaria in a pregnant woman: a case report. *Int. J. Infect. Dis.* 9 234–235. 10.1016/j.ijid.2004.08.002 15916911

[B29] Goldenberg R. Culhane J. Iams J. (2009). Preterm birth 1: epidemiology and causes of preterm birth. *Obstet. Anesth.* 371 75–84. 10.1111/j.1440-1754.2012.02536.x PMC7134569 18177778

[B30] Hall N. Marianna K. Raine J. D. Carlton J. M. Kooij T. W. A. Berriman M. (2005). A comprehensive survey of the plasmodium life cycle by genomic. transcriptomic, and proteomic analyses. *Science* 307 82–86. 10.1126/science.1103717 15637271

[B31] Heazell A. E. P. Moll S. J. Jones C. J. P. Baker P. N. Crocker I. P. (2007). Formation of syncytial knots is increased by hyperoxia. hypoxia and reactive oxygen species. *Placenta* 28 S33–S40. 10.1016/j.placenta.2006.10.007 17140657

[B32] Hioki A. Hioki Y. Ohtomo H. (1990). Influence of pregnancy on the course of malaria in mice infected with plasmodium berghei. *J. Protozool.* 37 163–167. 10.1111/j.1550-7408.1990.tb01121.x 2193152

[B33] Hviid L. Marinho C. R. F. Staalsoe T. Penha-Gonçalves C. (2010). Of mice and women: rodent models of placental malaria. *Trends Parasitol.* 26 412–419. 10.1016/j.pt.2010.04.010 20605743

[B34] Ismail M. R. Ordi J. Menendez C. Ventura P. J. Aponte J. J. Kahigwa E. (2000). Placental pathology in malaria: a histological, immunohistochemical, and quantitative study. *Hum. Pathol.* 31 85–93. 10.1016/S0046-8177(00)80203-80208 10665918

[B35] Janssen C. S. Phillips R. S. Turner M. R. Barret M. P. (2004). Plasmodium interspersed repeats: the major multigene superfamily of malaria parasites. *Nucleic Acids Res.* 32 5712–5720. 10.1093/nar/gkh907 15507685 PMC528792

[B36] Justice M. J. Dhillon P. (2016). Using the mouse to model human disease: increasing validity and reproducibility. *Dis. Model. Mech.* 9 101–103. 10.1242/dmm.024547 26839397 PMC4770152

[B37] Kanellopoulos-Langevin C. Caucheteux S. M. Verbeke P. Ojcius D. M. (2003). Tolerance of the fetus by the maternal immune system: role of inflammatory mediators at the feto-maternal interface. *Reprod. Biol. Endocrinol.* 1 1–6. 10.1186/1477-7827-1-121 14651750 PMC305337

[B38] Kraemer S. M. Smith J. D. (2006). A family affair: var genes, PfEMP1 binding, and malaria disease. *Curr. Opin. Microbiol.* 9 374–380. 10.1016/j.mib.2006.06.006 16814594

[B39] Lager S. Powell T. L. (2012). Regulation of nutrient transport across the placenta. *J. Pregnancy* 2012 1–14. 10.1155/2012/179827 PMC3523549 23304511

[B40] Lamb T. J. Brown D. E. Potocnik A. J. Langhorne J. (2006). Insights into the immunopathogenesis of malaria using mouse models. *Expert Rev. Mol. Med.* 8 1–22. 10.1017/S1462399406010581 16556343

[B41] Laochan N. Zaloumis S. Imwong M. Lek-Uthai U. Brockman A. Sriprawat K. (2015). Intervals to *Plasmodium falciparum* recurrence after anti-malarial treatment in pregnancy: a longitudinal prospective cohort. *Malar. J.* 14:221. 10.1186/s12936-015-0745-749 26017553 PMC4449611

[B42] Lawn J. E. Blencowe H. Waiswa P. Amouzou A. Mathers C. Hogan D. (2016). Stillbirths: rates, risk factors, and acceleration towards 2030. *Lancet* 387 587–603. 10.1016/S0140-6736(15)00837-835 26794078

[B43] Lima F. A. Gómez-Conde I. Videira P. A. Marinho C. R. F. Olivieri D. N. Tadokoro C. E. (2014). Intravital microscopy technique to study parasite dynamics in the labyrinth layer of the mouse placenta. *Parasitol. Int.* 63 254–259. 10.1016/j.parint.2013.06.012 23845789

[B44] Lucchi N. W. Sarr D. Owino S. O. Mwalimu S. M. Peterson D. S. Moore J. M. (2011). Natural hemozoin stimulates syncytiotrophoblast to secrete chemokines and recruit peripheral blood mononuclear cells. *Placenta* 32 579–585. 10.1016/j.placenta.2011.05.003 21632106 PMC3142316

[B45] Luxemburger C. Ricci F. Nosten F. Raimond D. Bathet S. White N. J. (1997). The epidemiology of severe malaria in an area of low transmission in Thailand. *Trans. R. Soc. Trop. Med. Hyg.* 91 256–262. 10.1016/S0035-9203(97)90066-3 9231189

[B46] Malvy D. Torrentino-Madamet M. L’Ollivier C. Receveur M. C. Jeddi F. Delhaes L. (2018). Plasmodium falciparum recrudescence two years after treatment of an uncomplicated infection without return to an area where malaria is endemic. *Antimicrob. Agents Chemother.* 62 1–5. 10.1128/AAC.01892-1817 29229635 PMC5786779

[B47] Marinho C. R. F. Neres R. Epiphanio S. Gonçalves L. A. Catarino M. B. Penha-Gonçalves C. (2009). Recrudescent *Plasmodium berghei* from pregnant mice displays enhanced binding to the placenta and induces protection in multigravida. *PLoS One* 4:e5630. 10.1371/journal.pone.0005630 19461965 PMC2680968

[B48] Mayor A. Serra-Casas E. Bardají A. Sanz S. Puyol L. Cisteró P. (2009). Sub-microscopic infections and long-term recrudescence of Plasmodium falciparum in Mozambican pregnant women. *Malar. J.* 8 1–10. 10.1186/1475-2875-8-9 19134201 PMC2633011

[B49] McCarthy R. Martin-Fairey C. Sojka D. K. Herzog E. D. Jungheim E. S. Stout M. J. (2018). Mouse models of preterm birth: suggested assessment and reporting guidelines†. *Biol. Reprod.* 99 922–937. 10.1093/biolre/ioy109 29733339 PMC6297318

[B50] Megnekou R. Hviid L. Staalsoe T. (2009). Variant-specific immunity to Plasmodium berghei in pregnant mice. *Infect. Immun.* 77 1827–1834. 10.1128/IAI.01321-1328 19237516 PMC2681742

[B51] Menendez C. (1995). Malaria during pregnancy: a priority area of malaria research and control. *Parasitol* 11 178–183. 10.1016/0169-4758(95)80151-0 15275350

[B52] Menendez C. Ordi J. Ismail M. R. Ventura P. J. Aponte J. J. Kahigwa E. (2000). The impact of placental malaria on gestational age and birth weight. *J. Infect. Dis.* 181 1740–1745. 10.1086/315449 10823776

[B53] Menéndez C. Romagosa C. Ismail M. R. Carrilho C. Saute F. Osman N. (2008). An autopsy study of maternal mortality in Mozambique: the contribution of infectious diseases. *PLoS Med.* 5:e44. 10.1371/journal.pmed.0050044 18288887 PMC2245982

[B54] Moore K. A. Simpson J. A. Wiladphaingern J. Min A. M. Pimanpanarak M. Paw M. K. (2017). Influence of the number and timing of malaria episodes during pregnancy on prematurity and small-for-gestational-age in an area of low transmission. *BMC Med.* 15:117. 10.1186/s12916-017-0877-6 28633672 PMC5479010

[B55] Moormann A. M. Sullivan A. D. Rochford R. A. Chensue S. W. Bock P. J. Nyirenda T. (1999). Malaria and pregnancy: placental cytokine expression and its relationship to intrauterine growth retardation. *J. Infect. Dis.* 180 1987–1993. 10.1086/315135 10558956

[B56] Murray S. A. Morgan J. L. Kane C. Sharma Y. Heffner C. S. Lake J. (2010). Mouse gestation length is genetically determined. *PLoS One* 5:e12418. 10.1371/journal.pone.0012418 20811634 PMC2928290

[B57] Muthusamy A. Achur R. N. Bhavanandan V. P. Fouda G. G. Taylor D. W. Gowda D. C. (2004). Plasmodium falciparum-infected erythrocytes adhere both in the intervillous space and on the villous surface of human placenta by binding to the low-sulfated chondroitin sulfate proteoglycan receptor. *Am. J. Pathol.* 164 2013–2025. 10.1016/S0002-9440(10)63761-63763 15161637 PMC1615783

[B58] Muthusamy A. Achur R. N. Valiyaveettil M. Botti J. J. Taylor D. W. Leke R. F. (2007). Chondroitin sulfate proteoglycan but not hyaluronic acid is the receptor for the adherence of Plasmodium falciparum-infected erythrocytes in human placenta, and infected red blood cell adherence up-regulates the receptor expression. *Am. J. Pathol.* 170 1989–2000. 10.2353/ajpath.2007.061238 17525266 PMC1899447

[B59] Neres R. Marinho C. R. F. Gonçalves L. A. Catarino M. B. Penha-Gonçalves C. (2008). Pregnancy outcome and placenta pathology in Plasmodium berghei ANKA infected mice reproduce the pathogenesis of severe malaria in pregnant women. *PLoS One* 3:e1608. 10.1371/journal.pone.0001608 18270595 PMC2229663

[B60] Nosten F. Rogerson S. J. Beeson J. G. McGready R. Mutabingwa T. K. Brabin B. (2004). Malaria in pregnancy and the endemicity spectrum: what can we learn? *Trends Parasitol.* 20 425–432. 10.1016/j.pt.2004.06.007 15324733

[B61] Oduola A. M. J. Holbrook T. W. Galbraith R. M. Bank H. Spicer S. S. (1982). Effects of malaria (*Plasmodium berghei*) on the maternal-fetal relationship in mice. *J. Protozool.* 29 77–81. 10.1111/j.1550-7408.1982.tb02883.x 7045348

[B62] Oduola A. M. J. Phillips J. H. Spicer S. S. Galbraith R. M. (1986). *Plasmodium berghei* - histology. immunocytochemistry, and ultrastructure of the placenta in rodent malaria. *Exp. Parasitol.* 62 181–193. 10.1016/0014-4894(86)90022-6 3527738

[B63] Olivier M. Van Den Ham K. Shio M. T. Kassa F. A. Fougeray S. (2014). Malarial pigment hemozoin and the innate inflammatory response. *Front. Immunol.* 5:25 10.3389/fimmu.2014.00025 PMC3913902 24550911

[B64] Ordi J. Ismail M. R. Ventura P. J. Kahigwa E. Hirt R. Cardesa A. (1998). Massive chronic intervillositis of the placenta associated with malaria infection. *Am. J. Surg. Pathol.* 22 1006–1011. 10.1097/00000478-199808000-00011 9706981

[B65] Ordi J. Menendez C. Ismail M. R. Ventura P. J. Palacín A. Kahigwa E. (2001). Placental malaria is associated with cell-mediated inflammatory responses with selective absence of natural killer cells. *J. Infect. Dis.* 183 1100–1107. 10.1086/319295 11237836

[B66] Othoro C. Moore J. M. Wannemuehler K. A. Moses S. Lal A. Otieno J. (2008). Elevated gamma interferon-producing NK cells, CD45RO memory-like T cells, and CD4 T cells are associated with protection against malaria infection in pregnancy. *Infect. Immun.* 76 1678–1685. 10.1128/IAI.01420-1427 18250175 PMC2292852

[B67] Parekh F. K. Davison B. B. Gamboa D. Hernandez J. Branch O. L. H. (2010). Placental histopathologic changes associated with subclinical malaria infection and its impact on the fetal environment. *Am. J. Trop. Med. Hyg.* 83 973–980. 10.4269/ajtmh.2010.09-0445 21036823 PMC2963955

[B68] Pathak G. Asnani P. J. Vinayak V. K. (1990). Effect of prior eradication of Plasmodium berghei infection on the foetal development and parasitaemic levels under the stress of pregnancy. *J. Hyg. Epidemiol. Microbiol. Immunol.* 34 139–146. 2212635

[B69] Pavia C. S. Niederbuhl C. J. (1991). Immunization and protection against malaria during murine pregnancy. *Am. J. Trop. Med. Hyg.* 44 176–182. 10.4269/ajtmh.1991.44.176 2012261

[B70] Poovassery J. Moore J. M. (2006). Murine malaria infection induces fetal loss associated with accumulation of *Plasmodium chabaudi* AS-infected erythrocytes in the placenta. *Infect. Immun.* 74 2839–2848. 10.1128/IAI.74.5.2839-2848.2006 16622222 PMC1459757

[B71] Poovassery J. Moore J. M. (2009). Association of malaria-induced murine pregnancy failure with robust peripheral and placental cytokine responses. *Infect. Immun.* 77 4998–5006. 10.1128/IAI.00617-619 19687196 PMC2772554

[B72] Poovassery J. S. Sarr D. Smith G. Nagy T. Moore J. M. (2009). Malaria-induced murine pregnancy failure: distinct roles for IFN-γ and TNF. *J. Immunol.* 183 5342–5349. 10.4049/jimmunol.0901669 19783682 PMC2772180

[B73] Rai P. Majumdar K. Sharma S. Chauhan R. Chandra J. (2015). Congenital malaria in a neonate: case report with a comprehensive review on differential diagnosis, treatment and prevention in Indian perspective. *J. Parasit. Dis.* 39 345–348. 10.1007/s12639-013-0342-341 26064034 PMC4456531

[B74] Robbins J. R. Bakardjiev A. I. (2012). Pathogens and the placental fortress. *Curr. Opin. Microbiol.* 15 36–43. 10.1016/j.mib.2011.11.006 22169833 PMC3265690

[B75] Rodrigues-Duarte L. De Moraes L. V. Barboza R. Marinho C. R. Franke-Fayard B. Janse C. J. (2012). Distinct placental malaria pathology caused by different *Plasmodium berghei* lines that fail to induce cerebral malaria in the C57BL/6 mouse. *Malar. J.* 11 1–9. 10.1186/1475-2875-11-231 22799533 PMC3485172

[B76] Rodrigues-Duarte L. Pandya Y. Neres R. Penha-Gonçalves C. (2018). Fetal and maternal innate immunity receptors have opposing effects in severity of experimental malaria in pregnancy: beneficial roles for fetal-derived TLR4 and IFNAR1. *Infect. Immun.* 86 IAI.708–IAI.717. 10.1128/IAI.00708-717 PMC5913849 29440369

[B77] Rogerson S. J. Desai M. Mayor A. Sicuri E. Taylor S. M. van Eijk A. M. (2018). Burden, pathology, and costs of malaria in pregnancy: new developments for an old problem. *Lancet Infect. Dis.* 18 e107–e118. 10.1016/S1473-3099(18)30066-30065 29396010

[B78] Rogerson S. J. Hviid L. Duffy P. E. Leke R. F. Taylor D. W. (2007). Malaria in pregnancy: pathogenesis and immunity. *Lancet Infect. Dis.* 7 105–117. 10.1016/S1473-3099(07)70022-70021 17251081

[B79] Rogerson S. J. Pollina E. Getachew A. Tadesse E. Lema V. M. Molyneux M. E. (2003). Placental monocyte infiltrates in response to Plasmodium falciparum malaria infection and their association with adverse pregnancy outcomes. *Am. J. Trop. Med. Hyg.* 68 115–119. 10.4269/ajtmh.2003.68.1.0680115 12556159

[B80] Rossant J. Cross J. C. (2001). Placental development: lessons from mouse mutants. *Nat. Rev. Genet.* 2 538–548. 10.1038/35080570 11433360

[B81] Rossant J. Tam P. P. L. (2017). New insights into early human development: lessons for stem cell derivation and differentiation. *Cell Stem Cell* 20 18–28. 10.1016/j.stem.2016.12.004 28061351

[B82] Salanti A. Staalsoe T. Lavstsen T. Jensen A. T. R. Sowa M. P. K. Arnot D. E. (2003). Selective upregulation of a single distinctly structured var gene in chondroitin sulphate A-adhering *Plasmodium falciparum* involved in pregnancy-associated malaria. *Mol. Microbiol.* 49 179–191. 10.1046/j.1365-2958.2003.03570.x 12823820

[B83] Sarr D. Bracken T. C. Owino S. O. Cooper C. A. Smith G. M. Nagy T. (2015). Differential roles of inflammation and apoptosis in initiation of mid-gestational abortion in malaria-infected C57BL/6 and A/J mice. *Placenta* 36 738–749. 10.1016/j.placenta.2015.04.007 25956987 PMC4466201

[B84] Sarr D. Smith G. M. Poovassery J. S. Nagy T. Moore J. M. (2012). Plasmodium chabaudi AS induces pregnancy loss in association with systemic pro-inflammatory immune responses in A/J and C57BL/6 mice. *Parasite Immunol.* 34 224–235. 10.1111/j.1365-3024.2012.01355.x 22251385 PMC3296870

[B85] Schofield L. Grau G. E. (2005). Immunological processes in malaria pathogenesis. *Nat. Rev. Immunol.* 5 722–735. 10.1038/nri1686 16138104

[B86] Sharma A. Conteh S. Langhorne J. Duffy P. E. (2016). Heterologous infection of pregnant mice induces low birth weight and modifies offspring susceptibility to malaria. *PLoS One* 11:e0160120. 10.1371/journal.pone.0160120 27467392 PMC4965193

[B87] Sharma L. Shukla G. (2014). Treatment of pregnant BALB/c mice with sulphadoxine pyrimethamine or chloroquine abrogates Plasmodium berghei induced placental pathology. *Parasitol. Int.* 63 49–56. 10.1016/j.parint.2013.08.016 24013006

[B88] Sharma L. Shukla G. (2017). Placental malaria: a new insight into the pathophysiology. *Front. Med.* 4:117. 10.3389/fmed.2017.00117 28791290 PMC5524764

[B89] Shulman C. E. Dorman E. K. Bulmer J. N. (2002). Malaria as a cause of severe anaemia in pregnancy. *Lancet* 360:494 10.1016/s0140-6736(02)09662-9 12241758

[B90] Silver K. L. Zhong K. Leke R. G. F. Taylor D. W. Kain K. C. (2010). Dysregulation of angiopoietins is associated with placental malaria and low birth weight. *PLoS One* 5:e9481. 10.1371/journal.pone.0009481 20208992 PMC2830425

[B91] Souza R. M. Ataíde R. Dombrowski J. G. Ippólito V. Aitken E. H. Valle S. N. (2013). Placental histopathological changes associated with plasmodium vivax infection during pregnancy. *PLoS Negl. Trop. Dis.* 7:e2071. 10.1371/journal.pntd.0002071 23459254 PMC3573078

[B92] Takao K. Miyakawa T. (2015). Genomic responses in mouse models greatly mimic human inflammatory diseases. *Proc Natl Acad Sci.* 112 1167–1172. 10.1073/pnas.1401965111 25092317 PMC4313832

[B93] Takata K. Fujikura K. Shin B.-C. (1997). Ultrastructure of the rodent placental labyrinth: a site of barrier and transport. *J. Reprod. Dev.* 43 13–24. 10.1262/jrd.43.13 9275091

[B94] Tegoshi T. Desowitz R. S. Pirl K. G. Maeno Y. Aikawa M. (1992). Placental pathology in plasmodium berghei-infected Rats. *Am. J. Trop. Med. Hyg.* 47 643–651. 10.4269/ajtmh.1992.47.643 1449205

[B95] Umbers A. J. Aitken E. H. Rogerson S. J. (2011). Malaria in pregnancy: small babies, big problem. *Trends Parasitol.* 27 168–175. 10.1016/j.pt.2011.01.007 21377424

[B96] van Hensbroek M. B. Palmer A. Onyiorah E. Schneider G. Jaffar S. Dolan G. (1996). The effect of a monoclonal antibody to tumor necrosis factor on survival from childhood cerebral malaria. *J. Infect. Dis.* 174 1091–1097. 10.1093/infdis/174.5.1091 8896514

[B97] van Zon A. A. J. C. Eling W. M. C. (1980). Pregnancy associated recrudescence in murine malaria (*Plasmodium berghei*). *Tropenmed. Parasitol.* 31 402–408. 7015633

[B98] van Zon A. A. J. C. Eling W. M. C. Hermsen C. C. (1985). Pregnancy-induced recrudescences strengthen malarial immunity in mice infected with *Plasmodium berghei*. *Parasitology* 91 9–17. 10.1017/s003118200005647x 3897957

[B99] van Zon A. A. J. C. Eling W. M. C. Hermsen C. C. R. Koekkoek A. A. G. M. (1982). Corticosterone regulation of the effector function of malarial immunity during pregnancy. *Infect. Immun.* 36 484–491. 10.1016/j.scitotenv.2015.10.076 7044972 PMC351253

[B100] Varo R. Crowley V. M. Sitoe A. Madrid L. Serghides L. Kain K. C. (2018). Adjunctive therapy for severe malaria: a review and critical appraisal. *Malar. J.* 17 1–18. 10.1186/s12936-018-2195-2197 29361945 PMC5781278

[B101] Vinayak V. K. Pathak G. Asnani P. J. Jain S. Malik A. K. (1986). Influence of malarial infection on the maternal-foetal relationship in pregnant mice. *Aust. J. Exp. Biol. Med. Sci.* 64 223–227. 10.1038/icb.1986.24 3533019

[B102] Walter P. R. Garin Y. Blot P. (1982). Placental pathologic changes in malaria: a histologic and ultrastructural study. *Am. J. Pathol.* 109 330–342. 6758604 PMC1916118

[B103] Wildman D. E. Chen C. Erez O. Grossman L. I. Goodman M. Romero R. (2006). Evolution of the mammalian placenta revealed by phylogenetic analysis. *Proc. Natl. Acad. Sci. U.S.A.* 103 3203–3208. 10.1073/pnas.0511344103 16492730 PMC1413940

[B104] World Health Organization (2018). *World Malaria Report 2018.* Geneva: WHO.

